# Navigating with grid and place cells in cluttered environments

**DOI:** 10.1002/hipo.23147

**Published:** 2019-08-13

**Authors:** Vegard Edvardsen, Andrej Bicanski, Neil Burgess

**Affiliations:** ^1^ Department of Computer Science NTNU—Norwegian University of Science and Technology Trondheim Norway; ^2^ Institute of Cognitive Neuroscience University College London, Alexandra House, 17 Queen Square, WC1N 3AZ London UK

**Keywords:** entorhinal cortex, grid cells, hippocampus, place cells, spatial navigation

## Abstract

Hippocampal formation contains several classes of neurons thought to be involved in navigational processes, in particular place cells and grid cells. Place cells have been associated with a topological strategy for navigation, while grid cells have been suggested to support metric vector navigation. Grid cell‐based vector navigation can support novel shortcuts across unexplored territory by providing the direction toward the goal. However, this strategy is insufficient in natural environments cluttered with obstacles. Here, we show how navigation in complex environments can be supported by integrating a grid cell‐based vector navigation mechanism with local obstacle avoidance mediated by border cells and place cells whose interconnections form an experience‐dependent topological graph of the environment. When vector navigation and object avoidance fail (i.e., the agent gets stuck), place cell replay events set closer subgoals for vector navigation. We demonstrate that this combined navigation model can successfully traverse environments cluttered by obstacles and is particularly useful where the environment is underexplored. Finally, we show that the model enables the simulated agent to successfully navigate experimental maze environments from the animal literature on cognitive mapping. The proposed model is sufficiently flexible to support navigation in different environments, and may inform the design of experiments to relate different navigational abilities to place, grid, and border cell firing.

## 1 INTRODUCTION

**Figure 1:**
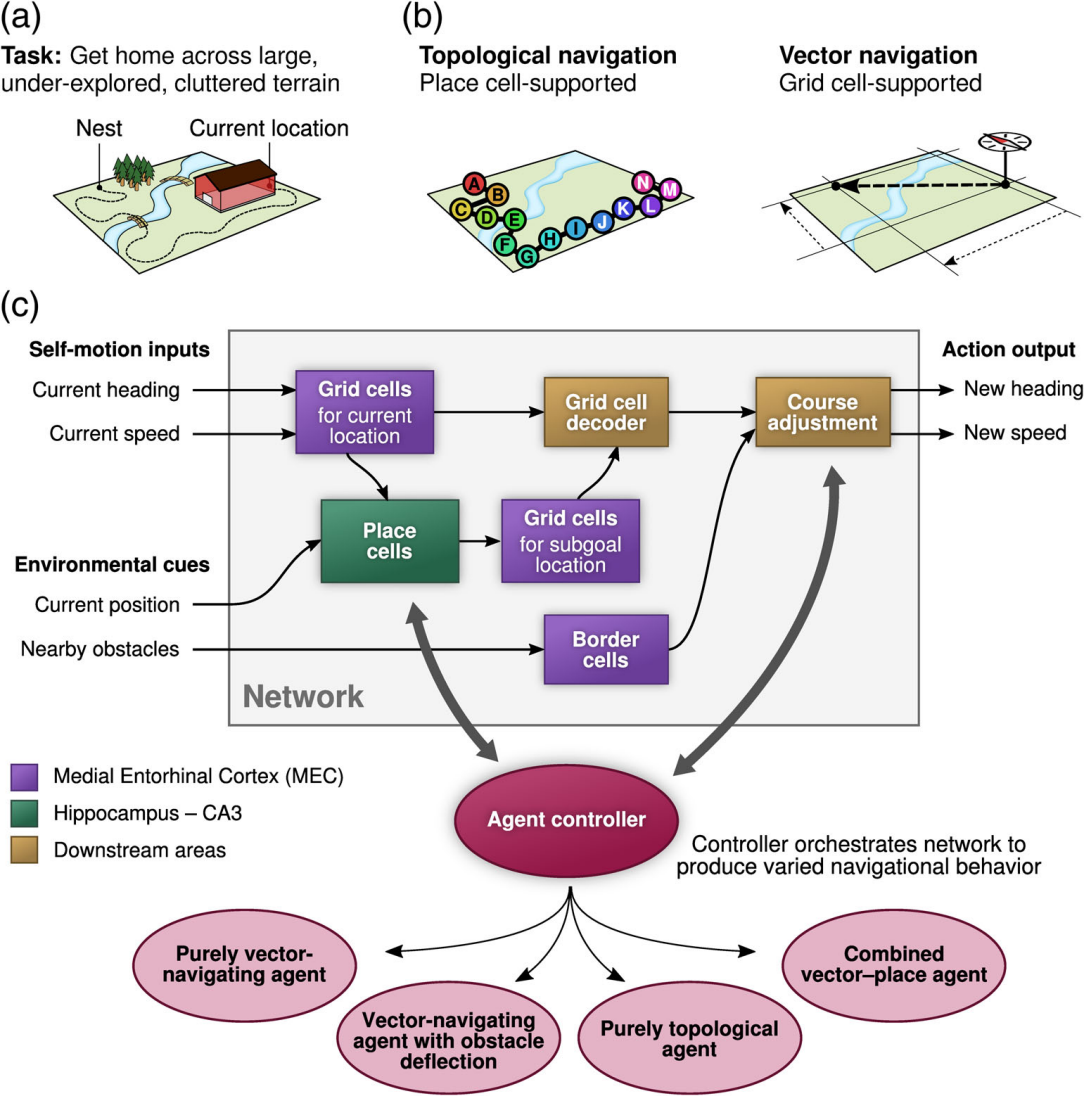
(a) Stereotypical navigation task. An agent has traveled across unknown terrain to a remote location and wishes to return to its nest, with limited knowledge of the environment. (b) Two major navigation paradigms supported by neurophysiological evidence. Place cells likely support topological navigation, where knowledge about locations' interconnectivity is used to reach the goal. Grid cells likely enable the calculation of distances and angles for straight‐line trajectories between arbitrary pairs of previously visited locations (vector navigation). (c) Model overview. Network portion (gray box) remains fixed across all trials. An external agent controller orchestrates components in the network in order to produce a variety of navigational strategies, either primarily vector‐based navigation, primarily topological navigation, or combined strategies utilizing a mixture of information from grid cells, place cells, and border cells. A grid cell decoder performs vector navigation toward a subgoal provided by the place cells. Border cells provide local obstacle information to a course adjustment mechanism. Box colors indicate related areas in the hippocampal formation [Color figure can be viewed at wileyonlinelibrary.com]

Successfully navigating the environment is a problem common to most animals. There are a wide range of approaches to navigation, mirroring the wide range of behavioral requirements across different species (Trullier, Wiener, Berthoz, & Meyer, 1997). In mammals, navigation is thought to be supported in part by a “cognitive map” (O'Keefe & Nadel, 1978), an internal neural representation of space. Such a map would endow an animal with navigational planning capabilities that should enable it to robustly find its way to previously visited locations (Figure 1a). The theoretical notion of the cognitive map is supported by compelling neurophysiological evidence. Hippocampal place cells represent unique locations in the environment (O'Keefe & Dostrovsky, 1971), and the more recently discovered grid cells in the medial entorhinal cortex (Hafting, Fyhn, Molden, Moser, & Moser, 2005) appear to provide a spatial metric by encoding the animal's coordinates in the two‐dimensional plane. The discoveries of head‐direction cells (Taube, Muller, & Ranck, 1990a, 1990b) and border cells/boundary vector cells (Barry et al., 2006; Lever, Burton, Jeewajee, O'Keefe, & Burgess, 2009; Solstad, Boccara, Kropff, Moser, & Moser, 2008) further strengthen the hypothesis of an internal neural map of space.

Place cells, particularly in the hippocampal area CA3, are thought to form interconnections through recurrent synapses such that neighborhood relationships between locations in the explored environment might be retrievable from the synaptic strengths between place cells. Such a system could implement a “topological navigation” strategy (Figure 1b), whereby the agent navigates to its goal location by calculating the shortest path in this internal representation of the environment and then following the resultant sequence of place cells' firing fields as its itinerary. Many models of place cell‐based navigation have emphasized this topological view, considering recurrent synapses among place cells to encode connectivity, distance, or transition probability between locations (Blum & Abbott, 1996; Gillner & Mallot, 1998; Mataric, 1991; Muller, Stead, & Pach, 1996; Redish & Touretzky, 1998; Stachenfeld, Botvinick, & Gershman, 2017).

Grid cells have been suggested to support goal vector representations (Bush, Barry, Manson, & Burgess, 2015; Edvardsen, 2015; Erdem & Hasselmo, 2012; Kubie & Fenton, 2012). Given grid cell activity for the present location and a trace of the grid cell activity for the goal location, the appropriate straight‐line vector across two‐dimensional space can be determined. The grid cell network can then support a “vector navigation” strategy (Figure 1b; Mittelstaedt & Mittelstaedt, 1980; Etienne, Maurer, & Séguinot, 1996), assuming that the allocentric goal vector can be transformed to the agent's egocentric frame of reference. Head‐direction cells and parietal gain field neurons (Pouget & Sejnowski, 1997; Snyder, Grieve, Brotchie, & Andersen, 1998) have been suggested to provide this function (Bicanski & Burgess, 2018; Burgess, Becker, King, & O'Keefe, 2001; Byrne, Becker, & Burgess, 2007). The agent can then find the correct bearing toward the goal location even across large stretches of unexplored space due to the metric properties of grid cells (Carpenter, Manson, Jeffery, Burgess, & Barry, 2015; Fiete, Burak, & Brookings, 2008).

According to these separate classes of computational model, place cells and grid cells seem to support complementary navigational strategies. Either strategy alone has its strengths and weaknesses: Place cell‐based topological navigation excels at finding the shortest possible paths needed to reach goals in cluttered and complicated environments, possibly including detours around known obstacles, but only if the agent has explored the environment extensively in advance, forming the necessary topological map. Conversely, grid cell‐based vector navigation can rely on goal vectors, even across long stretches of potentially unknown terrain, but obstacles along the straight‐line path to the goal might cause the agent to get stuck. A navigational strategy based on grid cells alone would not be sufficient outside of obstacle‐free open‐field environments, raising the question of whether or how grid cells participate in the navigation process under real‐world conditions.

Here we show how a grid cell‐based vector navigation model can be augmented to cope with environments cluttered by obstacles, based on the known aspects of hippocampal function. Medial entorhinal cortex (mEC) layer II, where grid cells are most prevalent, is a major input to the hippocampus, and the hippocampus in turn projects back to deeper layers of mEC. While the suggested interplay of grid cells and place cells has been modeled extensively at the circuit level, such work has usually focused on maintaining the firing properties of one population based on inputs from the other (Dordek, Soudry, Meir, & Derdikman, 2016; Hardcastle, Ganguli, & Giocomo, 2015; Kropff & Treves, 2008; Mulas, Waniek, & Conradt, 2016; Rolls, Stringer, & Elliot, 2006; Solstad, Moser, & Einevoll, 2006; Stachenfeld et al., 2017). Here we investigate how the distinct characteristics of these two representations of space can interact to guide behavior. We suggest a role for hippocampal replay events during navigation, using place cells to dynamically adjust the target for the vector navigation process, based on the intriguing possibility that place cells and grid cells can fire coherently during replay (Ólafsdóttir, Carpenter, & Barry, 2016). Additionally, the existence of border cells (Solstad et al., 2008) suggests that a grid cell‐supported vector navigation mechanism might have access to information about nearby obstacles, and hence the ability to make course adjustments based on their presence. Boundary vector cells (Barry et al., 2006; Lever et al., 2009) could serve a similar function, signaling boundary presence at a greater distance, forgoing the need for actual boundary contact before deflecting a trajectory. We combine these aspects of topological navigation and local obstacle avoidance with a grid cell‐based vector navigation model, and demonstrate that such an augmented vector navigation mechanism can efficiently navigate cluttered environments. The combined navigational strategy enables the agent both to negotiate complicated obstacles and to efficiently traverse long distances of unexplored space, potentially exploiting shortcuts.

## 2 MATERIALS AND METHODS

Here, we present the architecture and main features of the proposed hippocampal navigation model (Figure 1c), consisting of grid cells decodable to goal vectors, border cells for local obstacle avoidance, and a topological map implemented by place cells. Different types of obstacles present different challenges during navigation, and we describe how the model utilizes its components in concert to overcome these challenges. Grid cells, the grid cell decoder, and obstacle avoidance mechanism are represented by rate‐based neural networks in our implementation. The networks do not need any advance training, as the weights have been explicitly preconfigured for their intended roles in the model (see McNaughton, Battaglia, Jensen, Moser, and Moser (2006), Kubie and Fenton (2012) for how such grid cell networks might be obtained through learning). For simplicity, the place cell system is represented directly by a graph data structure, and the agent's high‐level control logic is represented by explicit rules. A more detailed description of the implementation is given in Supporting Information.

### 2.1 Grid cell decoding for vector navigation

At the core of the model—alongside place and border cells—is a set of grid cells together with a grid cell‐decoding mechanism. The main output of the network is the allocentric direction in which the agent should move next; this output is primarily driven by the grid cell decoder. The decoding mechanism confers vector navigation capabilities onto the model, by processing inputs from two separate grid cell populations and calculating the vector between the two respective locations represented by those populations. One of the grid cell populations encodes the agent's current location in the two‐dimensional plane, whereas the other population encodes the agent's destination. This arrangement is similar to previous work on vector navigation by grid cell decoding (Bush et al., 2015; Edvardsen, 2015), with one crucial extension: Here, the vector navigation destination does not necessarily have to be the same as the agent's ultimate goal location, but can change between different “subgoals” throughout the navigation process (see below).

Note that the model of combined vector and topological navigation proposed below is indifferent to the particular workings of the grid cell‐decoding mechanism, or indeed to the origin of the grid cell signal itself—we assume only that vector navigation can be performed through the readout of grid cells. Our specific implementation used here builds on the implementation from Edvardsen (2017), where grid cell decoding is performed according to a “nested” view of the grid cell system (Stemmler, Mathis, & Herz, 2015). Feedforward decoder neurons receive inputs from the two grid cell populations and are preconfigured to detect specific patterns of directional offset between the two inputs. A goal vector can be inferred in as few as one to two synapses (Edvardsen, 2018), thus even a fast sweep through multiple locations (e.g., within the timeframe of a replay event) can continuously update the goal vector. Assuming reasonably fast synaptic integration and neuronal time constants, a postsynaptic neuron could fire within 5–10 ms, which would allow for approximately 10 place fields to be sampled within a replay episode. In addition, replay events (chained together; Davidson, Kloosterman, & Wilson, 2009) can last longer than 120 ms, potentially covering longer trajectories, suggesting that the order of magnitude estimate for the timescales in the model is consistent with published data.

The grid cells are implemented as a set of recurrent neural networks (specifically, continuous attractor networks; Burak & Fiete, 2009), which perform path integration on a self‐motion velocity input in order to maintain an updated grid cell representation. Although no noise is explicitly added to this process, some drift may nevertheless occur over time due to imperfect path integration by the grid cell networks. Within the context of the presented simulations the drift is negligible, though future versions of the model could be extended to utilize sensory inputs for error correction in the grid cell system.

### 2.2 Place cells learn a topological map

Place cells, particularly in the hippocampal area CA3, have been suggested to represent space as a graph structure by virtue of their synaptic interconnections (Muller et al., 1996; Redish & Touretzky, 1998). For simplicity we implemented the place cells directly as nodes in a place graph. New place nodes are created whenever the agent is sufficiently far away from the place field of any previously created place node, that is, the agent instantaneously memorizes novel locations. Each node takes a snapshot of the grid cell ensemble's current activity, effectively establishing a link between a given place cell and the grid cells for later coordinated replay.

Next, bidirectional links are formed between pairs of place cell nodes whenever the agent moves from the place field of one node to another, corresponding to one‐shot Hebbian learning between traversed place cells. The resulting place cell graph reflects the topology of the explored environment and contains sufficient information to calculate the shortest paths between arbitrary pairs of start and goal place cells across those explored parts; the graph can, for example, determine which of the current immediately adjacent place fields lies on the shortest path to the destination. By always moving toward the neighboring place cell located on that shortest path, the agent would implement a topological navigation behavior. We implemented a graph search algorithm directly using the place graph structure, but we assume that the hippocampal place cell system can support a similar search mechanism, for example, via the resistive network of recurrent synapses (Muller et al., 1996).

### 2.3 Combining topological and vector navigation

Topological navigation does not require grid cells, as navigation is always directed toward a local place cell. However, combining a topological map with grid cell decoding yields additional navigational capabilities. We assume that an active place cell can trigger the reinstatement of the corresponding grid cell activity for the corresponding location, possibly mediated by projections from CA1/subiculum to medial entorhinal cortex (Bush, Barry, & Burgess, 2014; van Strien, Cappaert, & Witter, 2009). In the model, each place node is associated with a snapshot of the grid cell activity at the time of the place cell's creation. By associating each visited place field with its unique grid cell activity pattern, any location can become the start or end point for vector navigation. This enables powerful combinations of vector navigation and topological navigation and can help an agent overcome obstacles by selecting a more suitable place field as its subgoal. We propose that hippocampal replay events can be used to sample possible subgoals among place cell firing fields to allow the agent to change its current destination (see below).

Hence, the model accommodates purely topological, purely vectorial, and combined navigational strategies within the same network architecture. Throughout the navigation process, the relative impact of each strategy is influenced by external factors (bold arrows in Figure 1c) that may affect the strength of obstacle deflection (see below), trigger new hippocampal replay events, or induce periods of random exploration (see the Supporting Information).

### 2.4 Negotiating obstacles via border cells

Grid cell‐derived goal vectors do not account for any obstacles that might lie in the direct path. To steer the agent clear of such obstacles, we employ border cell signals as inputs to a course adjustment mechanism. This obstacle avoidance mechanism can be sufficient to overcome some of the obstacles encountered by the agent, but—due to the local nature of the information conveyed by the border cells—there will inevitably be certain obstacles that are insurmountable to it.

**Figure 2:**
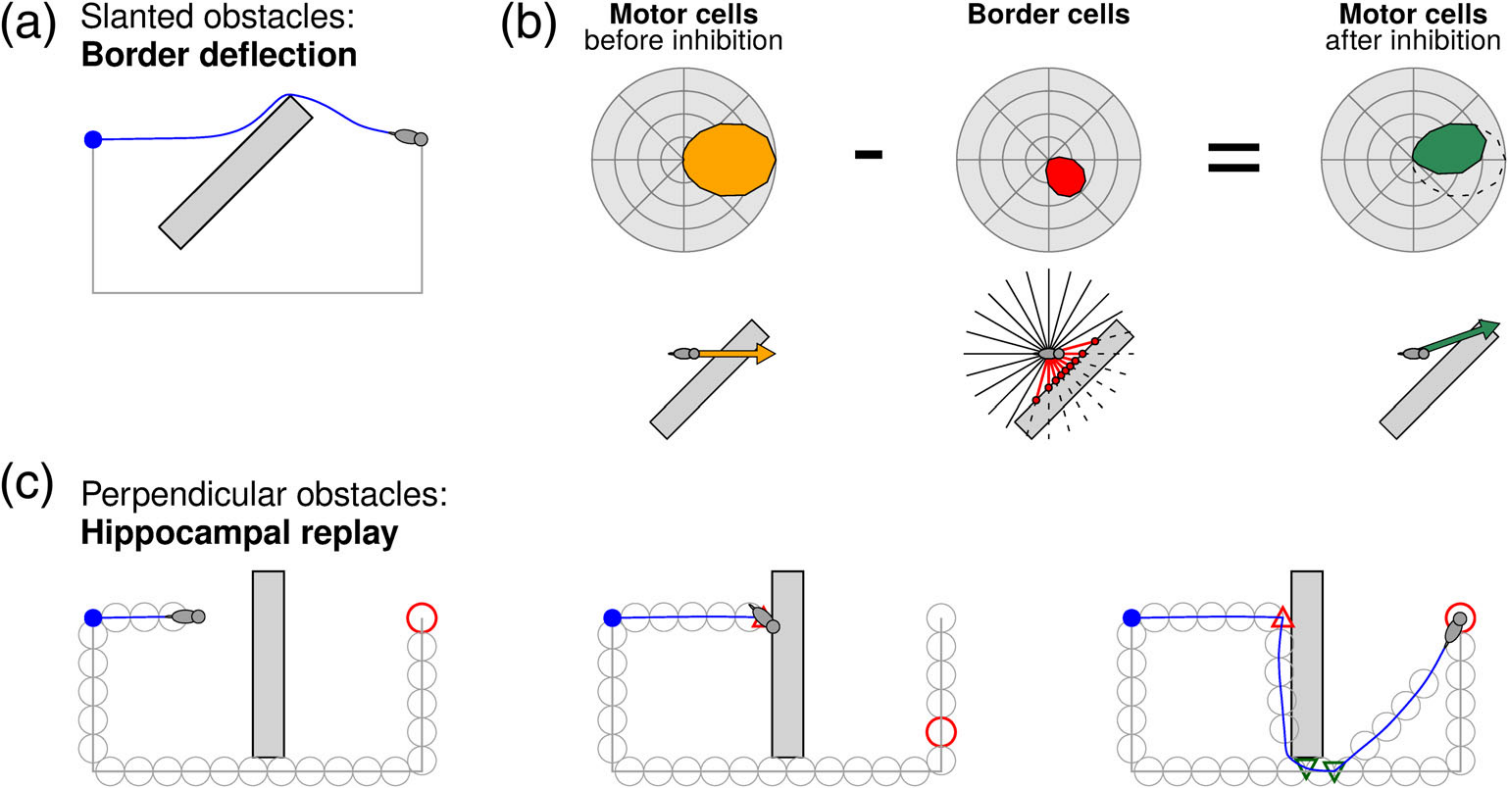
(a) Obstacles that form a slanted angle (less than 90°) with the goal vector can be negotiated by deflecting the direction of motion away from the obstacle. Goal distance keeps decreasing along the diverted trajectory. (b) Obstacle deflection mechanism: A bump of activity is induced in a ring of motor cells, pointing in the direction of the goal vector decoded from the grid cells. Each border cell responds to nearby obstacles in a particular allocentric direction and inhibits the corresponding motor cell, causing the population vector readout of the motor cells to steer away from the obstacle. (c) Perpendicular obstacles have no direction in which to successfully deflect the agent's motion—upon reaching a perpendicular obstacle (first panel), a new vector navigation subgoal must thus be selected. A hippocampal replay event is initiated at the goal location and propagated toward the current location, while concurrently updating the goal vector (second panel; red circle shows location of replay event). When a new viable destination is found, the agent diverts there and performs topological navigation for a while, before eventually resuming vector navigation (third panel; see Figure 3 for legend) [Color figure can be viewed at wileyonlinelibrary.com]

Obstacles that form a slanted angle with the goal vector (less than 90°) can be avoided by deflecting the agent's direction of motion away from the obstacle, all the while remaining on a course that brings it closer to the goal, as the deflected vector still points less than 90° away from the true goal direction (Figure 2a). Since the goal vector is continuously updated (see the description of decoder in Section 2.1), the vector will continue to point to the target as the agent is deflected by obstacles. The agent can follow the deflected vector until either of two events occurs: The obstacle has been cleared, in which case the agent can resume navigating along the true goal vector, or the agent has followed the slope of the obstacle to a point where the border now forms a perpendicular obstacle to the goal vector and the deflection mechanism fails. Note that some obstacles might technically be perpendicular to the goal vector yet be trivially negotiable by small amounts of random exploration, such as a cylindrical shape encountered head‐on. We treat these among the slanted obstacles and use the term “perpendicular” throughout to refer to obstacles that remain a problem even after limited random exploration (e.g., a partially concave shape). In these cases, the agent selects a new subgoal via replay (see the next section).

The deflected movement vector (distinct from the vector derived from grid cell decoder) is calculated by ring networks of “motor cells” (resembling head‐direction cells), which combine the true goal direction decoded from grid cells with obstacle information from border cells (Figure 2b; see also Supporting Information). The border cells each respond to obstacles in a particular allocentric direction, with a stronger activity as distance decreases, and inhibit corresponding motor cells with the same allocentric tuning direction (see Burgess, Recce, and O'Keefe (1994) for a similar approach). The population vector readout of the motor cell population will then tend to steer away from the obstacle (Figure 2b, rightmost panel). When the agent is initially far away from an obstacle, the inhibition, and thus the deflection, is barely noticeable. However, because of rapid growth in border cell inhibition, the deflection increases in strength as the obstacle is approached—resulting in a trajectory that gently curves away from the obstacle (Figure 2a; Video S1).

The goal vector is constantly updated to reflect the detour undertaken by the agent during the deflected trajectory. For example, whereas the goal vector originally pointed due East in the example in Figure 2a, after deflecting to the Northern corner of the obstacle the goal is now located in a Southeasterly direction. Because the grid cell decoder can always recalculate the correct goal direction from any potentially novel location along the deflected trajectory, the agent remains able to find the goal.

### 2.5 Selecting new subgoals through hippocampal replay events

Faced with “perpendicular obstacles” (locally perpendicular boundaries where random exploration is unable to trigger further progress), the local obstacle deflection mechanism will fail to find a viable path forward. Motor cells will be equally inhibited on either side of the goal vector, so there is no remaining direction of “least resistance” toward which to deflect the agent, which is now stuck. The agent must then select a new location as the currently active subgoal for the vector navigation process. We propose that this takes place through *hippocampal replay events*, which have been reported to occur when navigating animals stop at choice points or otherwise come to rest during maze sessions. These events are characterized by quick bursts of hippocampal neural activity that appear to play back, or “replay,” traces of earlier place cell activity along paths previously traveled—possibly remote from the animal's current location (Foster & Wilson, 2006; Ji & Wilson, 2007; Ólafsdóttir, Bush, & Barry, 2018). Intriguingly, simultaneously recorded grid cells have been reported to activate in coherence with the replaying place cells (Ólafsdóttir et al., 2016), suggesting that the grid cell population might mirror the replay trajectory by recalling the corresponding spatial locations of the reactivating place cells. As the grid cell decoder can infer a goal vector in one to two synapses (Edvardsen, 2018), this suggests that the (sub‐)goal vector can follow along on the timescale of the replay events. These would then be replay events where grid cells follow a replay in the place cell population through hippocampal–entorhinal projections (Ólafsdóttir et al., 2016). However, note that temporal coding phenomena may also arise in the grid cell population independently of the hippocampus (Hafting, Fyhn, Bonnevie, Moser, & Moser, 2008; O'Neill, Boccara, Stella, Schoenenberger, & Csicsvari, 2017).

Whenever the agent gets stuck, a replay event originating at the goal location and propagating toward the current location could thus be used to find candidates for the new subgoal. The agent initially tries to reach the ultimate goal location, but if the goal vector is blocked by an insurmountable obstacle, the subgoal shifts step by step along a replay trajectory—in our model the shortest path according to the place cell graph (that is, among all previously visited locations)—toward the current location. As soon as a place cell is encountered for which the grid cell‐decoded goal vector points sufficiently clear of any obstacles (that is, allowing the motor cells to activate despite inhibition from border cells), the agent resumes moving in that direction. The agent will thus initially prefer to navigate toward locations close to the goal location, attempting to quickly find shortcuts across open space using the grid cells. However, if all of these locations are blocked, the agent will eventually resort to finding its way back via a previously visited place cell close to the current location. Figure 2c and Video S1 show examples of the process during navigation; when the agent reaches the wall, a replay event propagates along the chain of place cells until a feasible subgoal past the obstacle has been found, and the agent then diverts there.

Since the replay events occur across place cells contained in the place graph, the choice of subgoal is restricted to previously visited locations—however, shortcuts can be taken due to the grid cell system. Once a subgoal has been reached, the agent follows a topological navigation strategy for a while, in order to ensure that it escapes the catchment area of the obstacle. Otherwise—if the agent immediately reverted to vector navigation—it might risk running back into the same obstacle. The agent eventually resumes vector navigation, to enable more potential shortcut discovery later in the trial. The duration of this topological navigation phase is governed by a configurable “resetting” probability (Supporting Information). Also note that, should a replay event propagate all the way to the current place cell—indicating that none of the place cells activated earlier during the replay were accepted as the new subgoal—we consider the path forward to be blocked. The agent will then unlearn the connection to the most recently activated place cell (the one across the blocked gap), so that the place graph again correctly reflects the topology of the environment.

## 3 RESULTS

Here, we present simulation results from our combined vector/topological navigation model, first demonstrating successful navigation in cluttered environments, next addressing key characteristics of environments where our combined navigation model is particularly well‐suited, and finally demonstrating how the model is sufficiently flexible to solve certain experimental mazes from the literature.

### 3.1 Navigating cluttered environments

**Figure 3:**
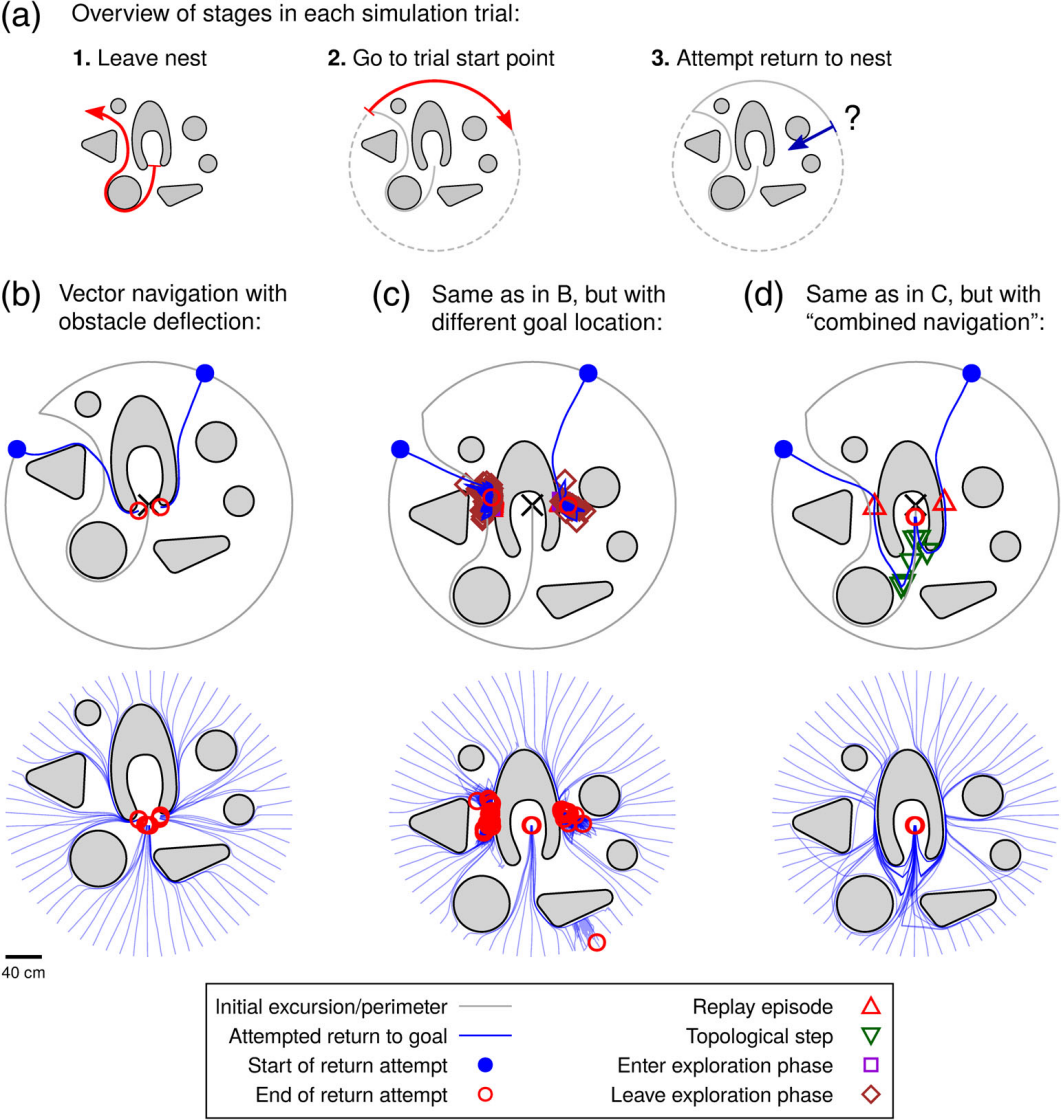
(a) Overview of trial stages. The agent leaves the nest (black cross in b/c/d) along a predefined trajectory for the initial exploration of the environment, performs a partial traversal of a perimeter in order to reach its prespecified starting position (all trials equally spaced across the full perimeter), and then attempts to return to the nest. (b) Results from 64 trials in an environment with a diverse set of obstacles, using a strategy of vector navigation with border cell‐based obstacle deflection. Upper panel shows two example trials from the set of simulations, while lower panel shows all trajectories from the full set of 64 trials, superimposed in the same plot. (c) Results from 64 trials with the same agent strategy as in (b), but with the environment modified so that the nest is now located further into the cave‐like structure. This creates perpendicular obstacles where the agent gets stuck. (d) Results from 64 trials in the same environment as in (c), but now using the combined vector–place strategy that can utilize the topological map to select a new subgoal whenever stuck [Color figure can be viewed at wileyonlinelibrary.com]

Figure 3a shows an example of the kinds of environments employed to test the simulated agent's navigational abilities. Large open spaces are interspersed with obstacles of various shapes centered around a “nest” location in the middle of the arena. Each trial consists of a training phase and a test phase, with the agent initially located at the nest location without any pre‐existing knowledge of the environment. The agent first follows a given outbound path from the nest, while updating grid cell and place cell population activity as usual during this initial excursion (performing path integration in the grid cells, generating new place cells in the place cell graph, associating them with the contemporary grid cell population snapshot, and forming links among place cells when moving from one field to the next). It then moves along a perimeter around the environment to a given starting location, before navigating back to the nest. These starting locations are spread out along the perimeter in order to assess the robustness of the agent's navigational ability under different conditions.

Simulation results from augmenting pure vector navigation with an obstacle deflection mechanism are presented in Figure 3b. Two example trials show the agent successfully returning to the nest location from two different starting points along the circular perimeter (Figure 3b, upper panel), and the results from all 64 trials superimposed in one plot shows that the agent is indeed successful in reaching the nest location from all tested starting points (Figure 3b, lower panel). The obstacle deflection mechanism allows the agent to locally deflect away from obstacles that lie ahead in its vector toward the goal, in this case enabling it to navigate all the way back to the goal. Whether boundary deflection alone is sufficient or not for successful navigation is determined by environmental characteristics; if all encountered obstacles present slanted surfaces (i.e., appear convex to an agent heading toward the goal; Figure S2A in Supporting Information), then this form of navigation will succeed.

An environment with only slanted obstacles will be the exception rather than the norm. Even in the favorable situation discussed above, the situation looks quite different if we move the nest just a short distance into the surrounding “cave” structure. Figure 3c shows results from a new set of trials with the nest in this changed location. A majority of the trials are no longer successful in reaching the goal, and most of the trials end with the agent stopping at two seemingly unremarkable locations along the outer cave wall. The obstacle forms a perpendicular border against the goal vector in these locations (i.e., appears concave to an agent heading toward the goal; Figure S2B). When the agent finds itself in these locations, practically all motor cell activity gets canceled by inhibition from border cells in the direction of the goal. In this situation, the agent initiates a brief period of random exploration before resuming attempted navigation toward the goal. However, as the goal distance increases on both sides of the perpendicular point (Figure S2B), the agent always ends up back at the same obstacle. These trials eventually expired after a timeout of 100 simulated seconds.

To avoid these failures, the agent diverts toward a different subgoal when halted by a perpendicular obstacle. Figure 3d presents results where the agent employs our proposed “combined” approach of augmenting vector navigation with replay‐based selection of a subgoal when stuck. A new subgoal is selected from the place graph by gradually shifting the subgoal closer to the current location until the decoded goal vector is no longer blocked by the obstacle. In the two example trajectories singled out in more detail, red triangular markers indicate the locations in which the agent gets stuck on a perpendicular obstacle and has to initiate a replay episode to find a new subgoal—each trajectory can be seen to continue onwards from the replay location on a diverted course toward a new subgoal. The “combined vector–place navigation” strategy is successful in guiding the agent away from perpendicular obstacles and ultimately to the final goal location, from all tested starting locations. See Video S2 for animated examples of trials performed as in Figure 3.

### 3.2 Advantages in sparsely explored environments

**Figure 4:**
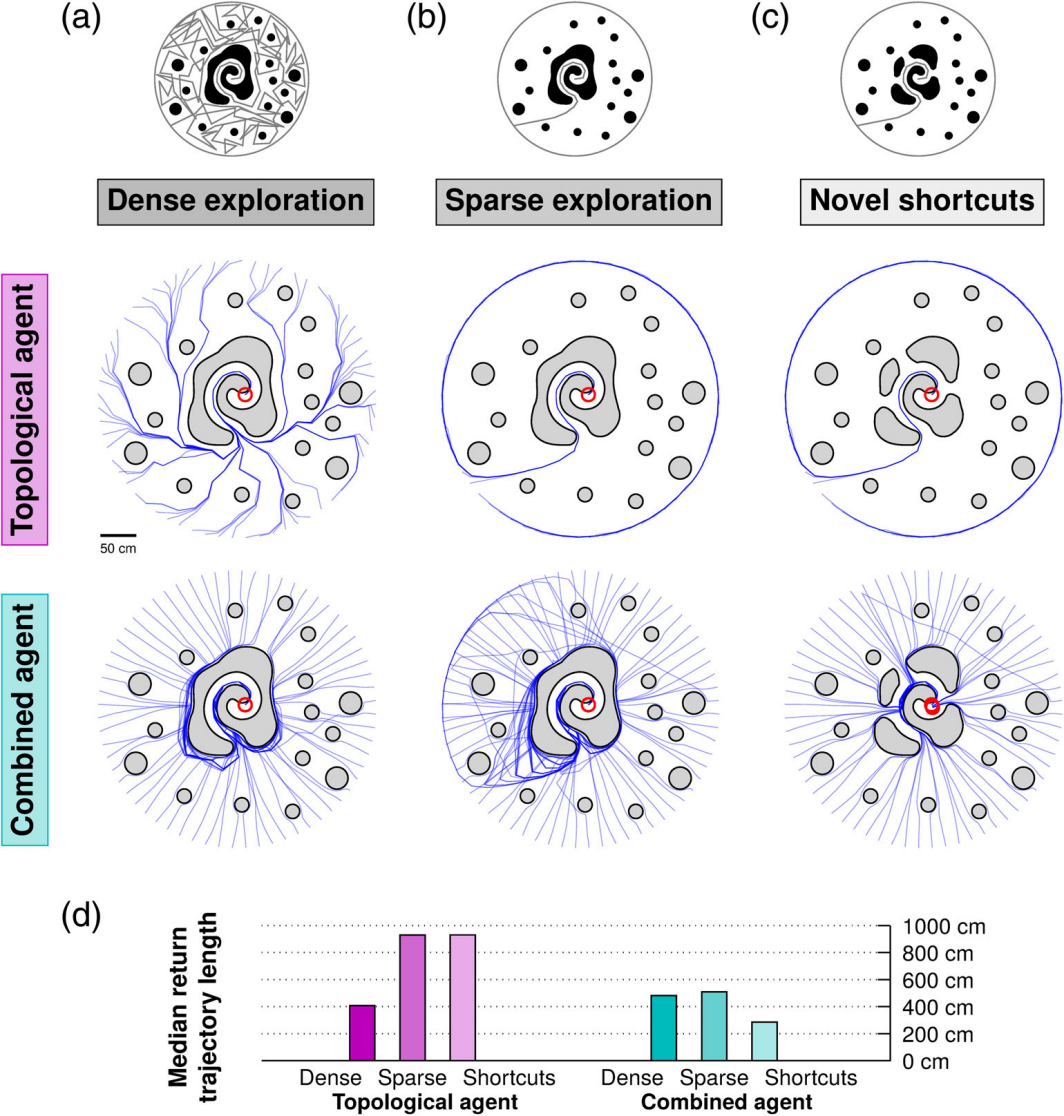
Comparing purely topological and combined vector–place navigation strategies. (a) Results from a densely explored environment. Upper panel shows 64 trials using a purely topological strategy, while lower panel shows 64 trials using the combined vector–place strategy. (b) As in (a), but now with only sparse exploration of the environment. (c) As in (b), but now with novel shortcuts introduced between the training and test phases. (d) Median return lengths for each set of trials shown in (a)–(c) [Color figure can be viewed at wileyonlinelibrary.com]

To compare the combined navigational strategy with a purely topological strategy, we used environments that had been densely explored (Figure 4a), sparsely explored (Figure 4b), or had novel shortcuts introduced after the training phase (Figure 4c). A quantitative comparison of the agent's navigational behavior across these configurations is depicted in Figure 4d, showing the median length of the paths needed to return to the goal location across the 64 trials for each unique configuration.

Dense versus sparse exploration refers to how many different parts of the environment the agent had visited before the navigation trial. For dense exploration, the agent's pre‐programmed exploration trajectory was drawn to cover close to the full environment with place fields (Figure 4a, top row), in order to facilitate a place graph with more nodes and connections. To quantify the difference in strategies, we considered close to full exploration of the environment versus only one outbound trajectory. However, there is no fixed amount of “necessary” exploration for the model to work. The proposed mechanism for selecting subgoals can be engaged with any amount of exploration, though subgoals must necessarily coincide with a previously visited location.

When the environment is densely explored, the purely topological navigation strategy is able to immediately follow the shortest paths around obstacles, without first having to run into them (Figure 4a). However, because the agent only navigates according to its learnt place cell graph, sparse knowledge of the environment results in suboptimal paths (Figure 4b), and it does not utilize any of the novel shortcuts introduced to the environment after the training phase (Figure 4c). The combined vector–place agent performs less well than the topological agent in the densely explored environment (Figure 4a), but performs better in the sparsely explored scenario (Figure 4b). The grid cell‐provided vector navigation capability enables the agent to shortcut across the open space not initially explored, and also to discover the novel shortcuts introduced after the training phase (Figure 4c). Because the combined agent's behavior is mostly driven by vector navigation until the later stages of the navigation trials, there is not much difference in performance between the densely and sparsely explored situations in this condition. See Video S3 for animated examples of trials performed as in Figure 4.

### 3.3 Flexibility to solve a variety of experimental mazes

We tested the navigation model in experimental environments from the animal navigation literature. Although relatively simple, we found that the flexible architecture underlying the navigation model made it possible to solve certain experimental tasks with minimal peripheral changes to the agent.

**Figure 5:**
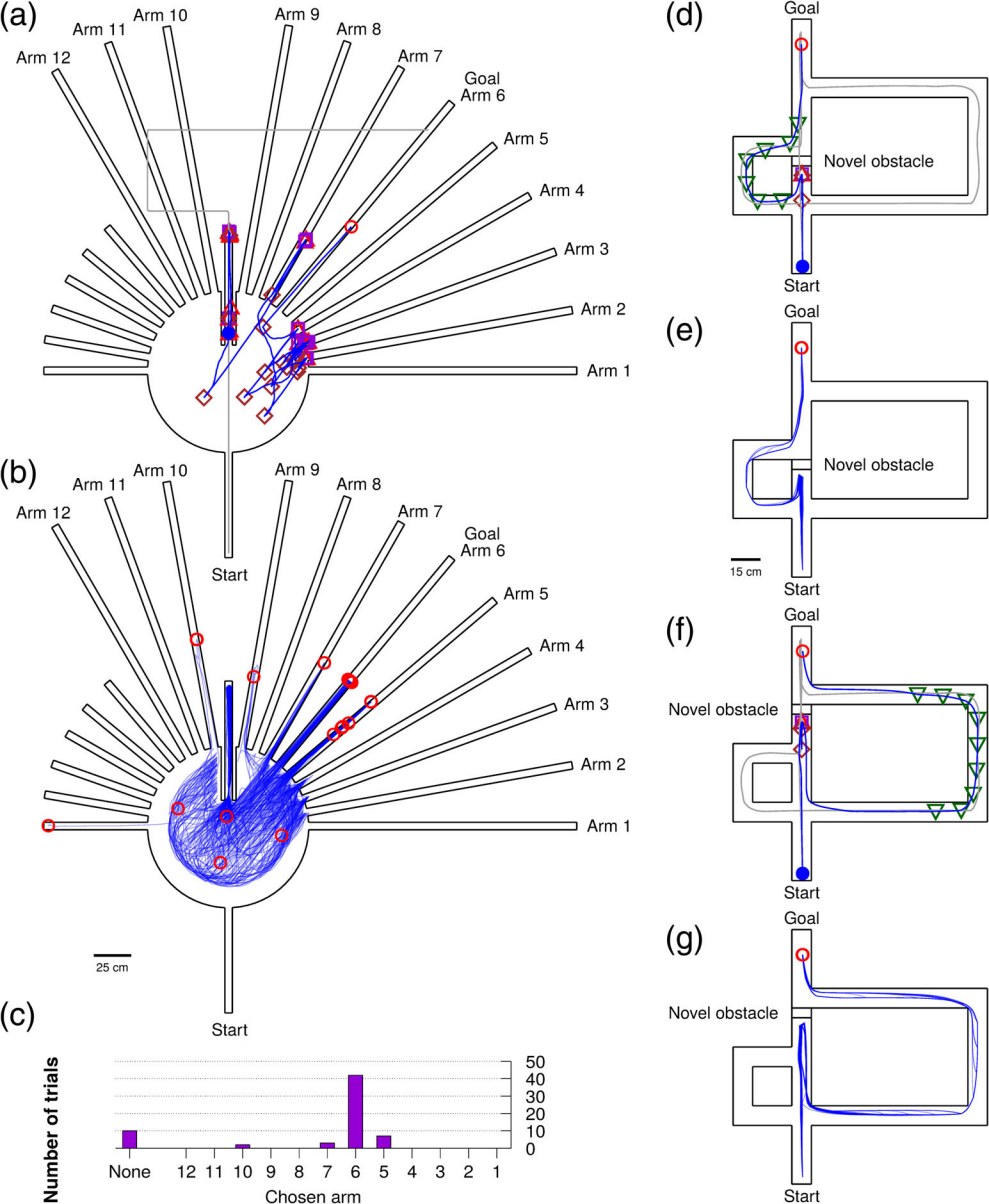
(a) A single example trial from a set of 64 trials in the sunburst maze, showing alternation between vector navigation attempts and random exploration that ultimately succeeds in finding the correct corridor. (b) Superimposed trajectories from the full set of trials in the sunburst maze. (c) Sunburst maze trials accumulated by outcome. (d) A single example trial from a set of 32 trials in the detour maze with the short corridor as the correct choice. (e) Superimposed trajectories from the full set of trials in the short version of the detour maze. (f) A single example trial from a set of 32 trials in the detour maze with the long corridor as the correct choice. (g) Superimposed trajectories from the full set of trials in the long version of the detour maze [Color figure can be viewed at wileyonlinelibrary.com]

An early inspiration for development of the cognitive map theory was the *sunburst maze* (Tolman, Ritchie, & Kalish, 1946), in which rats deduced the correct corridor toward a goal location despite major modifications to the environment between the initial training sessions and the test session. Specifically, the circuitous outbound corridor available during training (the gray line showing the agent's initial excursion in Figure 5a) was removed before the test session and replaced with a set of novel radial arms. Tolman et al. (1946) reported that 19 of 56 rats eventually chose the arm pointing directly toward the food location (arm 6), and hence demonstrated an ability to calculate the correct shortcut toward the goal. Though the methodology of this specific experiment has been challenged and their reported results have been difficult to reproduce (Bennett, 1996; Gentry, Brown, & Kaplan, 1947; Grieves & Dudchenko, 2013), we nevertheless wanted to see whether the navigation abilities claimed of the rats in this study were realizable within the framework of our navigation model.

To let our agent avoid the incorrect arms in the sunburst maze, we tune the motor cells in the boundary deflection mechanism to have a narrower width of their tuning curves. This causes the agent to abort its vector navigation‐driven goal approach whenever the current goal vector is misaligned with the angle of the current corridor, that is, whenever following the vector would make the agent run into corridor walls. The agent will then turn around to perform a period of random exploration, followed by another attempt to vector navigate toward the goal. Eventually, the agent might find itself in a favorable starting location where it has a clear path toward the goal location—the vector navigation process can then succeed in guiding the agent down the correct corridor without getting interrupted by the obstacle avoidance mechanism. This process of alternating between random exploration and vector navigation is visible in an example of a successful trial in Figure 5a (note that the rats in Tolman et al. (1946) on average used around three and a half minutes to select an arm). Superimposed results from the full set of 64 trials are shown in Figure 5b. Trials were terminated after a timeout of 100 simulated seconds or when the agents ventured a certain distance down a corridor (approximately corresponding to the length of the short arms; Tolman et al. (1946) also allowed rats to explore the initial segments of the arms). The majority of trials ended with the agent choosing the correct corridor (Figure 5c).

While the sunburst maze lends itself to a vector navigation‐based solution, an environment that might instead favor topological navigation is the detour maze (Alvernhe, Save, & Poucet, 2011; Tolman & Honzik, 1930). This maze consists of a direct corridor between the start location and the goal location, as well as two detour corridors that branch off near the starting location—a short detour and a long detour. The direct corridor can be blocked in one of two locations, so that either both detours can reach the goal, or only the long detour can reach the goal. A cognitive map should enable the animal to choose the shortest possible detour, depending on the location at which the novel obstacle is encountered. Simulations are shown in Figure 5d–g. When the agent encounters the novel obstacle, the place cell connection across the obstacle is severed and the chain of place cells linking the current location and the goal location down the main corridor is thus interrupted. The new shortest path in the place graph, along which the next replay event will propagate, then guides the agent toward the correct corridor in both the short detour scenario (Figure 5e) and the long detour scenario (Figure 5g). See Video S4 for animated examples of trials performed as in Figure 5.

## 4 DISCUSSION

We have presented a hippocampal navigation model that is able to navigate in cluttered environments by utilizing a combination of grid cell‐driven vector navigation, place cell‐driven topological navigation, and border cell‐driven local obstacle avoidance. The proposed architecture, which maps well onto known anatomy and electrophysiology of the hippocampal formation, can support a diverse range of navigational strategies by allowing external modulation of network components to produce different navigational behaviors. The agent initially performs vector navigation, primarily driven by grid cells and aided by border cells for obstacle deflection. If progress is blocked by obstacles, the agent initiates hippocampal replay that introduces aspects of topological navigation into the agent's overall behavior, allowing the agent to switch between different subgoals in order to successfully navigate complex environments. Our results demonstrate that grid cell decoders (Bush et al., 2015; Edvardsen, 2015; Stemmler et al., 2015) can be the primary driver of navigational processes even beyond the open‐field environment, because such vector navigation mechanisms can indeed work in cluttered environments when aided by place cells and border cells to negotiate obstacles.

Such a combined navigational strategy can be particularly useful in large, under‐explored environments (which applies to most natural, open environments), where the agent would otherwise have to resort to a topological strategy of navigating by retracing its original steps back to the origin. The grid cell‐based vector navigation process can instead guide the agent across novel territory, eventually resorting to a place cell‐driven topological navigation process should vector navigation turn out to be impossible. Besides potentially enabling more efficient navigation in underexplored scenarios, the combined strategy is useful for discovering preexisting or novel shortcuts in the environment (Figure 4). The agent is a “pragmatist,” trying out the fastest route first (a straight line). The hybrid aspect of the model (interacting with place cells for subgoal selection via replay events) is only engaged when it gets stuck. In experimental environments, animals often bias their exploration trajectories differently (Kubie & Fenton, 2009), for example, spending more time near walls, which could affect navigational performance, for example, restricting the availability of subgoals. However, to the best of our knowledge the tendency of experimental animals to spend more time near the walls is likely due to perceived safety and not navigational considerations. In larger, open, and underexplored environments, the pragmatic approach proposed here may be a simple, nondemanding, yet effective strategy for shortcut discovery and quick return to the nest. Future work should systematically investigate simplicity (i.e., pragmatism) versus optimality trade‐offs, which, however, will depend heavily on the structure of the environments used to assess optimality.

The flexibility of our proposed architecture is demonstrated by the model's performance in two examples of experimental maze environments from the animal navigation literature, namely the sunburst maze and the detour maze. Interestingly, we found that—while both types of maze might be cited as examples of animals expressing cognitive map‐based navigation capabilities—these two environments primarily exercised complementary parts of our navigation model. That is, the nature of these mazes is such that mostly only the vector navigation capacity or the topological navigation capacity of the model is utilized. Specifically, for the sunburst maze, only vector navigation with obstacle avoidance and random exploration was needed—the topological map was not used. On the other hand, in the detour maze, vector navigation would not strictly be necessary—the maze layout is fully known by the animal in advance, so navigating only according to the topological map should be sufficient (Martinet, Sheynikhovich, Benchenane, & Arleo, 2011). If the cognitive map is considered to consist of both the topological aspects of place cells and the metric aspects of grid cells, then experimental environments should ideally be designed to engage the aspects of the navigational circuit intended to be probed by the experimenter.

Besides this more general conclusion about navigational strategies and experimental environments, the model also makes certain predictions about the nature of hippocampal replay events. Replay has been suggested to be involved in both planning and consolidation (Carr, Jadhav, & Frank, 2011; Diba & Buzsáki, 2007; Foster & Wilson, 2006; Girardeau, Benchenane, Wiener, Buzsáki, & Zugaro, 2009; Karlsson & Frank, 2009; Wilson & McNaughton, 1994). Here we considered a potential role for replay in the planning of a trajectory (Pfeiffer & Foster, 2013). These events are triggered in the model whenever the agent gets stuck during the vector navigation process—we hence predict a higher propensity for replays to occur when the agent encounters obstacles. Replays should be coherent between place cells and grid cells (Ólafsdóttir et al., 2016). Whenever the agent diverts its course, the new destination should be in the vicinity of a recent replay, and there should be a goal vector representation (Sarel, Finkelstein, Las, & Ulanovsky, 2017) for this subgoal. The agent might follow different bearings across the same open field, depending on whether it encountered any obstacles earlier in the trial that caused it to change subgoals. In general, the model suggests that the analysis of behavioral and neurophysiological data might benefit from taking into account the location of obstacles and likely subgoals as relevant variables, not just the animal's own location and its ultimate destination. However, there seems to be a diversity of different forward/reverse replay/preplay phenomena (for review, see Ólafsdóttir et al., 2018), and the model proposed here only considers one type of replay.

The integration of grid cells and place cells in the same architecture for navigational purposes has been proposed before. Erdem and Hasselmo (2012, 2014) present a model for grid cell‐based vector navigation that depends on place cells as a critical component of the system. The model relies on the grid cells “simulating” hypothetical forward trajectories in different directions in order to trigger the activation of the target place cell and thus to have detected the correct goal direction. That is, it exploits projections from grid cells onto place cells to determine the goal direction, whereas the grid cell decoder in our model produces its goal vector through direct readout of the grid cell population (Bush et al., 2015; Edvardsen, 2015; Stemmler et al., 2015). Whereas Erdem and Hasselmo (2012, 2014) perform subgoal selection by diffusing a reward signal throughout the topological graph of place cells and navigating in the direction of the place cell most strongly activated by a simulated forward trajectory, we propose that hippocampal replay events might interact with a grid cell decoder for the same purpose.

In reinforcement learning parlance, both aspects of the current model fall into the category of model‐based approaches to navigation. A full model of rodent navigation should, in addition, contain interactions with (e.g., striatal) reinforcement learning mechanisms (Chavarriaga, Strösslin, Sheynikhovich, & Gerstner, 2005; Chersi & Burgess, 2015; Dollé, Sheynikhovich, Girard, Chavarriaga, & Guillot, 2010), and/or possibly a mechanism akin to the successor representation (Dayan, 1993; Gershman, 2018; Stachenfeld et al., 2017). A successor representation is comparable to the topological place cell representation used in the current model, and replay events could similarly propagate through it, selecting new subgoals. The recent theoretical framework where grid cells form a low‐dimensional state representation (obtained via an eigenvector decomposition of place cells; Dordek et al., 2016; Stachenfeld et al., 2017) can in principle identify bottleneck states in environments (e.g., doorways), though it is currently unclear how this computation would be carried at the level of neurons. Such states could also constitute interesting subgoals, but the eigenvector decomposition requires a thorough exploration of the environment, contrary to the present model. With regard to spatial navigation, other strategies such as taxis and landmark‐based navigation (Trullier et al., 1997) are also known to guide an animal's behavior, and should be incorporated for a more complete navigation model. Finally, look‐ahead and mental navigation could also interact with the combined vector–place strategy proposed here (Bicanski & Burgess, 2018; Erdem & Hasselmo, 2012, 2014). In mental navigation, simulated motion (potentially driven by mock motor efference and conveyed by grid cells; Bellmund, Deuker, Schröder, & Doeller, 2016; Horner, Bisby, Zotow, Bush, & Burgess, 2016) can be thought of as accompanied by a reinstatement of sensory representations bound to locations (via place cells) along the imagined trajectory (Bicanski & Burgess, 2018), and would hence be particularly useful if planning involves particular sensory aspects along the route.

In conclusion, we have shown that grid cells can potentially be used to drive navigation and shortcut discovery even in cluttered environments, if aided by place cells and border cells. Realistic navigational strategies in cluttered, large, and underexplored environments will likely utilize combinations of both vector navigation and topological navigation. Environments commonly used in animal navigation research may not exercise both of these systems at the same time. Designing experiments with underexplored parts of the environment could shed more light on the interplay between vector and topological strategies in animal behavior and lead to new insights in the role of grid cells and place cells in navigation. A flexible navigation system with a plausible neural implementation might also be of interest to the field of biologically inspired robotics, to enable robots to navigate according to biologically inspired principles in cluttered, underexplored environments.

## CONFLICT OF INTEREST

The authors declare no potential conflict of interest.

## Supporting information


**Data S1**: Supporting Information

Video S1

Video S2

Video S3

Video S4
