# Supplementary material for: Navigating with grid and place cells in cluttered environments
Source: Hippocampus. 2019 Aug 13;30(3):220–32. doi: 10.1002/hipo.23147 (PMC8641373; doi:10.1002/hipo.23147)
Supplement: Supplementary file 1 — Data S1: Supporting Information [file HIPO-30-220-s004.pdf]

Supplementary Materials – Edvardsen et al. (2019)

Navigating with grid and place cells in cluttered environments

Contents

1 Detailed model description **1**

1.1 Model inputs 1

1.2 Modulatory signals 3

1.3 Grid modules 3

1.4 Place graph 3

1.5 Motor output 4

2 Energy landscapes for vector navigation with obstacle deflection **4**

1 Detailed model description

Fig. S1 shows a more detailed version of the navigation model's schematic from the main paper, now including the specific modulatory signals used by the agent controller to orchestrate network behavior. The model executes 1000 timesteps per simulated second. The agent moves with a fixed simulated speed of 20 $^{\mathrm{cm}}/_{\mathrm{s}}$ (unless halted, see Sec. 1.5). On each timestep, the model components update in the following order: (1) Model inputs, including border cells, (2) modulatory signals, (3) grid modules, (4) place graph, and (5) motor output, labeled "course adjustment" in Fig. S1. Each step is described in more detail next.

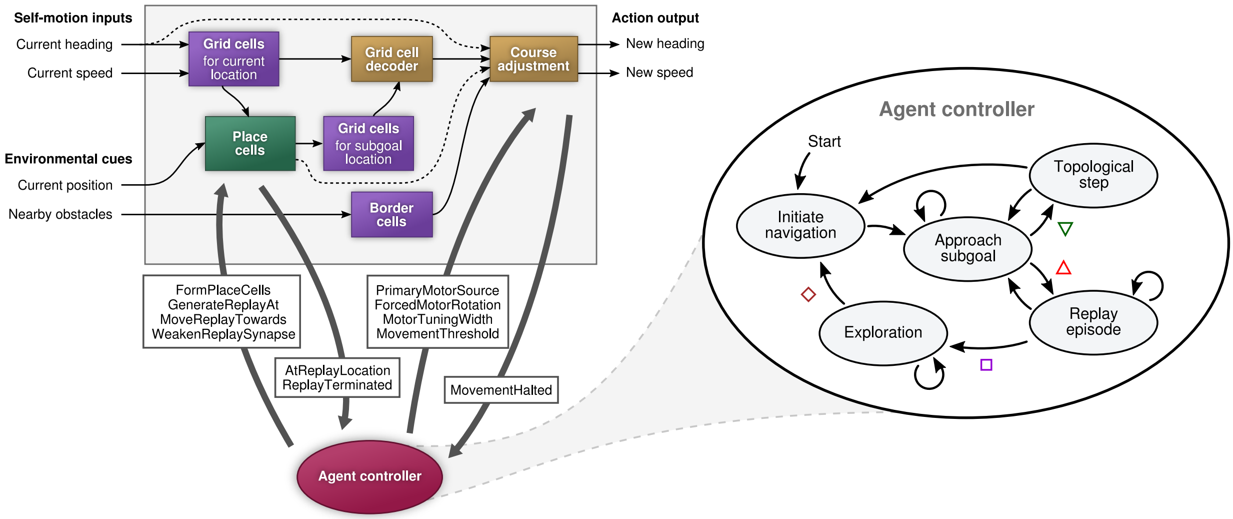

Figure S1: Detailed model schematic, including the modulatory signals used by the agent controller to orchestrate network behavior. The internal logic of the agent controller is organized as a "state machine", with distinct states and transitions between them. Symbols next to the state transition arrows correspond to the symbols used in the figures in the main paper. Dashed inputs to the course adjustment component indicate alternative sources for motor direction, see Sec. 1.5 for details.

1.1 Model inputs

In this step, besides updating velocity and x, y position inputs to the model, information about nearby obstacles is conveyed as follows: There are 72 border cells, each responsible for a particular allocentric direction equally spaced across 360°. For each border cell, a beam emanating from the agent's current location and extending 0.25 m in the cell's specified direction is tested for intersections with obstacles in the environment. If there were no intersections, the border cell gains an activation value of 0, otherwise the closest intersection distance d (in meters) is mapped through $2 \cdot e^{-5 \cdot d / 0.25}$ to get the final activation value for the border cell. In our implementation we used the Boost.Geometry library (`https://www.boost.org/`) to detect intersections.

Table S1: Pseudocode specification of the internal logic of the agent controller.

Default values reset before each timestep:	**FormPlaceCells** $\leftarrow \rho$<br>**GenerateReplayAt** $\leftarrow \emptyset$<br>**MoveReplayTowards** $\leftarrow \emptyset$<br>**WeakenReplaySynapse** $\leftarrow$ False<br>**PrimaryMotorSource** $\leftarrow$ GridCellDecoder<br>**ForcedMotorRotation** $\leftarrow 0$<br>**MotorTuningWidth** $\leftarrow \delta_{approach}$<br>**MovementThreshold** $\leftarrow \eta_{approach}$
Agent logic for state **InitiateNavigation**:	If ϕ is False, then **GenerateReplayAt** $\leftarrow$ GoalNode,<br>else { **GenerateReplayAt** $\leftarrow$ AgentNode; **MoveReplayTowards** $\leftarrow$ GoalNode. }<br>**NextState** $\leftarrow$ ApproachSubgoal
Agent logic for state **ApproachSubgoal**:	If **AtReplayLocation**, then **NextState** $\leftarrow$ TopologicalStep,<br>else if **MovementHalted**, then { **MovementThreshold** $\leftarrow \eta_{replay}$; **NextState** $\leftarrow$ ReplayEpisode, }<br>else **NextState** $\leftarrow$ ApproachSubgoal.
Agent logic for state **TopologicalStep**:	**GenerateReplayAt** $\leftarrow$ AgentNode<br>**MoveReplayTowards** $\leftarrow$ GoalNode<br>If $U(0,1) < \varepsilon_{reset}$, then **NextState** $\leftarrow$ InitiateNavigation,<br>else **NextState** $\leftarrow$ ApproachSubgoal.
Agent logic for state **ReplayEpisode**:	If not **MovementHalted**, then **NextState** $\leftarrow$ ApproachSubgoal,<br>else if **ReplayTerminated**, then { **WeakenReplaySynapse** $\leftarrow$ True; **PrimaryMotorSource** $\leftarrow$ LastHeading; **ForcedMotorRotation** $\leftarrow \pi$; **NextState** $\leftarrow$ Exploration, }<br>else { **MotorTuningWidth** $\leftarrow \delta_{replay}$; **MovementThreshold** $\leftarrow \eta_{replay}$; **MoveReplayTowards** $\leftarrow$ AgentNode; **NextState** $\leftarrow$ ReplayEpisode. }
Agent logic for state **Exploration**:	**PrimaryMotorSource** $\leftarrow$ LastHeading<br>**MotorTuningWidth** $\leftarrow \delta_{exploration}$<br>**ForcedMotorRotation** $\leftarrow N(\mu = 0,\ \sigma = 0.02)$<br>If $U(0,1) < \varepsilon_{exploration}$, then **NextState** $\leftarrow$ InitiateNavigation,<br>else **NextState** $\leftarrow$ Exploration.

Table S2: Description of parameters and their default values.

Parameter	Default	Description
ρ	True	Whether to form new place cells
ϕ	False	Whether to navigate topologically
$\varepsilon_{exploration}$	0.003	Probability of exploration ending
ε_{reset}	0.05	Probability of subgoal resetting
$\delta_{approach}$	0.75	Motor tuning for vector navigation[1]
δ_{replay}	0.1	Motor tuning for replay episodes[1]
$\delta_{exploration}$	0.1	Motor tuning for exploration periods[1]
$\eta_{approach}$	0.05	Halt threshold for vector navigation[2]
η_{replay}	0.2	Halt threshold for replay episodes[2]

[1] Tuning width in motor cells; larger values give more lenient obstacle deflection, small values require clearer line of sight.
[2] Threshold for confidence value c, below which agent will halt.

Table S3: Different versions of the agent used.

Vector-navigating agent with deflection *(Fig. 3BC)*
$\rho =$ False, rest default

Purely topological agent *(Fig. 4ABC upper row)*
$\phi =$ True, rest default

Combined agent *(Fig. 3D, 4ABC lower row, 5DEFG)*
All parameters set to defaults

Combined agent, sunburst version *(Fig. 5AB)*
$\varepsilon_{exploration} = 0.0005$, $\delta_{approach} = 0.1$, rest default

Combined agent, exaggerated traits *(Fig. 2AC)*
$\varepsilon_{reset} = 0.25$, $\eta_{replay} = 0.9$, rest default

1.2 Modulatory signals

The agent controller—in order to orchestrate the behavior of components in the hippocampal formation network—decides appropriate values for the modulatory inputs that flow from the controller into the network. The schematic in Fig. S1 shows that the internal logic of the agent controller is represented by a "state machine", consisting of different states/phases of the navigational process and possible transitions between them. The internal logic of these states needs to monitor the agent's progress in order to make decisions—this information is mediated by the modulatory outputs that flow from the network to the controller.

On each timestep, all modulatory inputs are first reset to their default values according to Table S1 upper row. The agent's current state—determined by the value of NEXTSTATE, which starts out as "InitiateNavigation"—is then used to calculate the present values for the modulatory inputs according to the corresponding state definition in Table S1. $\mathrm{U}(0,1)$ samples a uniformly random value between 0 and 1, and $\mathrm{N}(\mu,\sigma)$ samples from a Gaussian distribution. Modulatory outputs used in these calculations retain their values from the end of the previous timestep, and the specific meaning of all the different modulatory input/output signals are described in more detail in their relevant sections below.

Parametrization of the state logic was used to achieve the different agent versions used throughout the paper—see Table S2 for a specification of these parameters and their default values, and Table S3 for adaptations relevant to the specific agent versions.

1.3 Grid modules

The grid cell system, as well as the grid cell decoder, builds on our implementation from earlier work (Edvardsen, 2017). The system consists of 12 grid modules, each implemented as a continuous attractor network based on the model by Burak and Fiete (2009). The smallest grid module exhibits a grid scale of around 0.2 m, and each succeeding grid module scales up by a fixed factor of 1.5 from the previous one. This geometric progression of grid modules is then decoded according to a nested view of the grid cell system (Edvardsen, 2015; Stemmler et al., 2015). However, our model does not depend on any particular aspects of the grid cell system/decoder besides the following:

- There is a mechanism that can generate/maintain grid cell activity to represent the current location/spatial coordinates
- Whenever a new place cell is generated, that cell may take a "snapshot" of the current state of the grid cell population
- The grid cell decoder is able to calculate the approximate direction from the current location to a previously visited location, whenever it is presented with one of these earlier snapshots of the grid cell population (provided by the place cell system)

The reader is refered to Edvardsen (2015, 2017) for a full description.

1.4 Place graph

The place graph updates according to the following steps:

1. Retrieve the place cell closest to the agent's current x, y coordinates as the *visited place cell*
2. If the center of the visited place cell is located farther away than twice the place field radius, and FORMPLACECELLS is true, then create a new place cell at the current coordinates and use that as the visited place cell instead. Take a snapshot of the current grid cell population and store it alongside the newly created place cell
3. If the visited place cell is different from last timestep's visited place cell, and these two cells are not already connected, then form a connection between the two cells with a strength of 2
4. If WEAKENREPLAYSYNAPSE is true, then decrease the connection strength between the *last replaying place cell* and the *second-to-last replaying place cell*, and delete the connection altogether if the connection strength reached 0
5. If GENERATEREPLAYAT is different from $\emptyset$, then update the *replay place cell* to be either the visited place cell or the goal place cell, depending on whether the value of GENERATEREPLAYAT is respectively "AgentNode" or "GoalNode"
6. If MOVEREPLAYTOWARDS is different from $\emptyset$:
    - Update the replay place cell to be the place cell one step closer in the graph to either the visited place cell or the goal place cell, depending on whether the value of MOVEREPLAYTOWARDS is respectively "AgentNode" or "GoalNode", according to Breadth-First Search
    - If the search failed to find a path in the graph, or if the replay place cell was already at the indicated target node for the graph search, then set the output signal REPLAYTERMINATED to true on the next timestep
7. If the agent's distance to the center of the replay place cell is no greater than the place field radius, then set the output signal ATREPLAYLOCATION to true on the next timestep

8. Send the grid cell snapshot associated with the replay place cell to the grid cell decoder as the "target" for vector navigation

The place field radius was set to 13 cm in Figs. 3 and 4, and 6.5 cm in Figs. 2 and 5.

1.5 Motor output

There are two consecutive *motor networks*. Each motor network consists of two stages:

1. **Generating motor activity:** Receive a directional input α and then generate a Gaussian bump of activity in a ring of 72 motor neurons, pointing in the given direction (with tuning width/standard deviation δ for the Gaussian function, and afterwards rescaling all activation values so the peak activation value in the network is 1)
2. **Inhibiting motor cells from border cells:** Target each motor neuron with inhibition from the corresponding border cell, and then threshold negative activation values to 0. The final directional output of the motor network is calculated from the population vector average of the 72 motor neurons

The tuning width δ_1 for the first motor network is set to MOTORTUNINGWIDTH. The directional input α_1 to the first motor network is determined as follows (possibly with an extra offset if FORCEDMOTORROTATION is different from 0):

- If PRIMARYMOTORSOURCE is "GridCellDecoder", update the grid cell decoder and set α_1 as follows:
  - If the agent's distance to the center of the replay place cell is within three times the place field radius, consider the subgoal to be within visible range and explicitly calculate α_1 as the true bearing to the center of the replay place cell
  - Otherwise, set α_1 to the direction indicated by the grid cell decoder
- If PRIMARYMOTORSOURCE is "LastHeading", set α_1 to the agent's current bearing

The directional input α_2 to the second motor network is the output from the first motor network, and $\delta_2 = 0.1$. The combination of two motor networks is used to alleviate potential errors, e.g. traveling head-on toward sharp corners, when the border cells might symmetrically inhibit the motor population on both sides of the bump; even after receiving border cell inhibition, the agent would still be headed directly toward the obstacle. By repeating the entire process in a duplicated circuit (with a narrower/stricter tuning width), the agent can be halted if the deflected goal vector is still on a collision course with the obstacle.

To determine whether to halt the agent or to allow it to move, we calculate a "confidence" value c as follows: Let s_n denote the strength of the motor signal in motor network n after border cell inhibition as a ratio of the corresponding strength before inhibition. "Strength" here refers to the length of the population vector. The confidence value is then calculated as $c = \sqrt{s_1 \cdot s_2}$. The agent is halted if c is less than MOVEMENTTHRESHOLD, otherwise it is allowed to move in the direction indicated by the second motor network. The output signal MOVEMENTHALTED is set accordingly.

Special-case handling is added throughout to avoid divisions by zero, should e.g. all motor activity be cancelled out by border cell inhibition or the grid cell decoder return a zero-length goal vector. The agent will halt in these situations.

2 Energy landscapes for vector navigation with obstacle deflection

In the main paper we make the distinction between slanted and perpendicular obstacles, the former being negotiable by vector navigation augmented by an obstacle deflection mechanism. This property of slanted obstacles becomes apparent if we consider the navigation problem from the perspective of an "energy landscape"—considering each position in the environment to have as its "energy" the distance to the goal location—with the objective of the agent being to minimize this energy. The energy landscape can be visually inspected by drawing the geometry of the environment with a "reversed-polar" transformation; rather than drawing a given straight line according to its Euclidean coordinates, points along the line are transformed into goal direction θ and goal distance r—on respectively the x-axis and the y-axis—using the following expressions (varying t between 0 and 1):

$$\theta(t) = \text{atan2}\left(y_{start} + t \cdot (y_{end} - y_{start}),\; x_{start} + t \cdot (x_{end} - x_{start})\right),$$
$$r(t) = \sqrt{[x_{start} + t \cdot (x_{end} - x_{start})]^2 + [y_{start} + t \cdot (y_{end} - y_{start})]^2}.$$

In this transformed view of the environment, locations farther away from the goal—those with greater energy—are drawn higher on the y-axis, while the goal location—with zero energy—coincides with the zero level on the y-axis. Fig. S2A shows the results from Fig. 3B in the main paper transformed in this fashion. All trajectories start out along a horizontal line high in the energy landscape, because all instances of the agent were equally distant from the goal when they set out from their respective starting locations along the circular perimeter. The trajectories then proceed down along the y-axis toward the goal; vertical lines show trajectories that were able to progress directly toward the goal location unimpeded by obstacles, while encounters with slanted obstacles cause deflections to the left or right. Importantly, there are no "local minima" in this energy landscape—whenever the

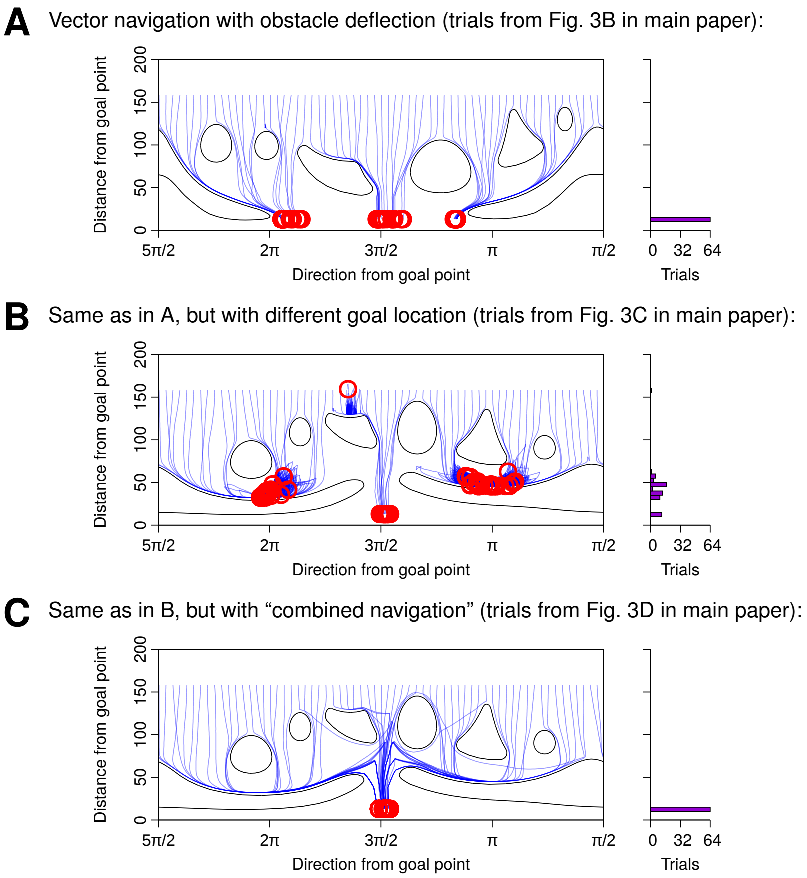

Figure S2: (A–C) Trials from Fig. 3B–D in the main paper, but with obstacles and trajectories plotted according to a “reversed-polar” transformation—making it possible to visually inspect where local minima will be in the environment given the particular goal location. Histograms of goal distances at trial termination show how many trials were successful in reaching the goal.

agent reaches an obstacle, there is always a deflected direction in which the goal distance keeps decreasing, and so the agent can remain on its downward trajectory through the energy landscape toward the goal location at the bottom.

Choosing a different goal location will alter the energy landscape; sometimes these changes will “warp” the landscape in such a way that the agent might become stuck where it previously would not, and vice versa. Fig. S2B shows the energy landscape after the goal location was moved in Fig. 3C in the main paper—local minima have now appeared, and the agent got stuck in these locations. However, this warping of the energy landscape—caused by moving the goal location—can also be used to the agent’s advantage: During hippocampal replay episodes in our model, the subgoal shifts gradually closer to the current location until the decoded goal vector is no longer blocked by the obstacle. At that point, the energy landscape has warped in such a way that the current agent location is no longer a local minimum, and the agent can therefore make further progress toward the goal. In Fig. S2C (from Fig. 3D in main paper), using the combined strategy, the agent was able thus to escape the local minima.

References

Burak, Y. and Fiete, I. R. (2009). Accurate path integration in continuous attractor network models of grid cells. *PLoS Comput. Biol.*, 5(2).

Edvardsen, V. (2015). A passive mechanism for goal-directed navigation using grid cells. In *Proceedings of the European Conference on Artificial Life (ECAL) 2015*, pages 191–198.

Edvardsen, V. (2017). Long-range navigation by path integration and decoding of grid cells in a neural network. In *Proceedings of the International Joint Conference on Neural Networks (IJCNN) 2017*.

Stemmler, M., Mathis, A., and Herz, A. V. (2015). Connecting multiple spatial scales to decode the population activity of grid cells. *Sci. Adv.*, 1(11):e1500816.
